# Gap Junctions Link Regular-Spiking and Fast-Spiking Interneurons in Layer 5 Somatosensory Cortex

**DOI:** 10.3389/fncel.2017.00204

**Published:** 2017-07-17

**Authors:** Robert J. Hatch, G. Dulini C. Mendis, Kai Kaila, Christopher A. Reid, Steven Petrou

**Affiliations:** ^1^The Florey Institute of Neuroscience and Mental Health, The University of Melbourne Melbourne, VIC, Australia; ^2^Department of Mechanical Engineering, The University of Melbourne Melbourne, VIC, Australia; ^3^Department of Biosciences and Neuroscience Center (HiLife), The University of Helsinki Helsinki, Finland; ^4^Department of Medicine (RMH), The University of Melbourne Melbourne, VIC, Australia; ^5^ARC Centre of Excellence for Integrated Brain Function, The University of Melbourne Melbourne, VIC, Australia

**Keywords:** gap junctions, electrophysiology, interneurons, cortex, fast-spiking, regular-spiking

## Abstract

Gap junctions form electrical synapses that modulate neuronal activity by synchronizing action potential (AP) firing of cortical interneurons (INs). Gap junctions are thought to form predominantly within cortical INs of the same functional class and are therefore considered to act within discrete neuronal populations. Here, we challenge that view and show that the probability of electrical coupling is the same within and between regular-spiking (RS) and fast-spiking (FS) cortical INs in 16–21 days old mice. Firing properties of these two populations were distinct from other INs types including neurogliaform and low-threshold spiking (LTS) cells. We also demonstrate that pre-junctional APs can depolarize post-junctional neurons and increase the probability of firing. Our findings of frequent gap junction coupling between functionally distinct IN subtypes suggest that cortical IN networks are much more extensive and heterogeneous than previously thought. This may have implications on mechanisms ranging from cognitive functions to modulation of pathological states in epilepsy and other neurological disorders.

## Introduction

Gap junctions form electrical synapses in networks of neocortical and hippocampal inhibitory interneurons (INs) that are implicated in a range of higher cognitive functions by virtue of their impact on network synchrony (Gibson et al., 1999; Beierlein et al., 2000; Galarreta and Hestrin, 2001). Gap junctions are composed of a pair of connexin hemichannels located in opposing, pre- and post-junctional, cell membranes (Bennett and Zukin, 2004). While connexins are encoded by at least 20 genes, eight of which are expressed in the mammalian brain, Cx36 is the predominant form of gap junctions between cortical INs (Söhl et al., 1998; Condorelli et al., 2000; Rash et al., 2000; Venance et al., 2000; Degen et al., 2004). Current evidence suggests that gap junctions form preferentially within INs of the same functional class (Galarreta and Hestrin, 1999, 2002; Gibson et al., 1999; Venance et al., 2000; Szabadics et al., 2001; Gibson et al., 2005; Hu and Agmon, 2015). Gibson et al. (1999) and Galarreta and Hestrin (1999) presented data suggesting that within the neocortex, electrical synapses occur in distinct networks comprised of either fast-spiking (FS) or low-threshold spiking (LTS) INs but were only rarely seen to occur between these classes. This type of electrical IN connectivity is thought to be critical in forming IN class specific networks (Monyer and Markram, 2004) that enhance synchronicity and support the generation of oscillations that underlie higher cognitive functions. For example, FS INs are involved in the generation of gamma frequency (20–100 Hz) activity, the power of which is reduced in Cx36 knock out mice (Hormuzdi et al., 2001; Buhl et al., 2003). Furthermore, Cx36 knock out mice also exhibit impaired short-term spatial memory (Allen et al., 2011). Another class of IN coupled by gap junctions are regular spiking INs (Szabadics et al., 2001), which are involved in the generation of beta (12–30 Hz) activity that is related to voluntary controlled sensorimotor actions (Salmelin et al., 1995; Szabadics et al., 2001; Tamás et al., 2004; Roopun et al., 2006). This prevailing view is challenged by a smaller number of studies that show that gap junction coupling may not only be limited to INs of the same class can but also occur between INs of different classes (Gibson et al., 1999; Caputi et al., 2009). Such evidence challenges the idea of IN class specific network function and implies that more complex network structures are possible.

It is not clear why the majority of studies have failed to detect significant levels of between IN class gap junction coupling and this may be due to sampling biases inherent in the molecular and electrophysiological methodology. For instance, studies that have used genetically labeled IN subclasses, including mice that express GFP in parvalbumin positive (PV^+^) INs, to determine connectivity would obviously lead to bias towards identification of synapses between these INs only (Galarreta and Hestrin, 2002; Hu and Agmon, 2015). More subtle biases may arise due to different abundances of IN subclasses. For example, in layer 5 (L5) of the mouse cortex, FS PV^+^ INs are the most common IN subclass (Xu et al., 2010), and it is therefore not surprising that these are the INs that have frequently been described to be coupled by gap junctions (Galarreta and Hestrin, 1999, 2002; Gibson et al., 1999; Venance et al., 2000; Oláh et al., 2007; Hu and Agmon, 2015). In an attempt to address this question, we used random sampling with four-electrode (quad) patch-clamp recordings in a small volume of L5 in the mouse somatosensory cortex to map electrical connectivity of large numbers of FS and regular-spiking (RS) INs. We provide strong evidence that coupling between and within these IN classes is identical, highlighting potential unidentified roles of INs that couple distinct subclasses in diverse brain functions.

## Materials and Methods

### Experimental Animals

All experimental procedures in this study were conducted in accordance with the Prevention of Cruelty to Animals Act 1986, under the guidelines of the NHMRC Code of Practice for the Care and Use of Animals for Experimental Purposes in Australia and were approved by the Florey Neuroscience Institute Animals Ethics Committee. GAD67+ mice (Tamamaki et al., 2003) were genotyped using polymerase chain reaction (PCR) of tail DNA at postnatal day 7 (P7).

### Brain Slice Preparation

GAD67-GFP mice (P16 – P21, *n* = 18) were anesthetized with 2% isoflurane and decapitated. The brain was removed quickly and placed into an iced slurry of cutting solution consisting of (mM): 125 Choline-Cl, 2.5 KCl, 0.4 CaCl_2_, 6 MgCl_2_, 1.25 NaH_2_PO_4_, 26 NaHCO_3_, 20 D-glucose saturated with 95% O_2_ plus 5% CO_2_. Three hundred micrometer coronal cortical slices were cut on a vibratome (VT1200; Leica; Germany) for whole-cell patch-clamp experiments. Slices were incubated at room temperature for a minimum of 1 h in artificial cerebral spinal fluid (aCSF) consisting of (mM): 125 NaCl, 2.5 KCl, 2 CaCl_2_, 2 MgCl_2_, 1.25 NaH_2_PO_4_, 26 NaHCO_3_, 10 D-glucose, saturated with 95% O_2_ plus 5% CO_2_ before patching.

### Whole-Cell Patch-Clamp Electrophysiology

Slices cut from GAD67-GFP mice were transferred to a submerged recording chamber on an upright microscope (Slicescope Pro 1000; Scientifica, UK) and perfused (8–10 ml/min) with aCSF at 32°C. L5 cortical INs, located no further than 100 μm apart, were visually identified using fluorescence targeted patching with infrared-oblique illumination microscopy with a 40× water-immersion objective (Olympus, Japan) and a CCD camera (IEEE 1394; Foculus, Germany). Quad patch-clamp recordings were made in current clamp mode using PatchStar micromanipulators (Scientifica, UK) and Axon Multiclamp 700B patch-clamp amplifiers (MDS, USA). Data were acquired using pClamp software (v10; MDS, USA) with a sampling rate of 50 kHz and low pass Bessel filtered at 10 kHz (Digidata 1440a; Axon). Patch pipettes (4–7 MΩ; GC150F-10; Harvard Instruments; USA) pulled using a Flaming/brown micropipette puller (Model P-1000; Sutter Instruments; USA) were filled with a solution consisting of (mM): 125 K-gluconate, 5 KCl, 2 MgCl_2_, 10 HEPES, 4 ATP-Mg, 0.3 GTP-Na, 10 phosophocreatine and 10 EGTA (pH 7.22 and 292 mOsm). 6-cyano-7-nitroquinoxaline-2,3-dione (CNQX; 20 μM; Sigma-Aldrich, Castle Hill, NSW, Australia) was used to block α-amino-3-hydroxy-5-methyl-4-isoxazolepropionic acid (AMPA) receptor-mediated currents.

### Electrophysiological Protocols

Once whole-cell configuration was obtained, a holding current was injected to maintain a membrane potential of approximately −70 mV and current steps were applied to characterize firing. To be included in the present study, a cell had to have an access resistance of less than 20 MΩ and a holding current of less than −200 pA throughout the entire recording. Electrical coupling was identified in cells based on post-junctional responses to pre-junctional current steps (−60 pA, 100 ms), where the time course and amplitude of the post-junctional voltage response indicated DC-coupling.

Two protocols were used to probe the impact of gap junction coupling on neuronal excitability using current clamp recordings. The effect of gap junction mediated pre-junction activity on post-junctional excitability was first investigated by injecting an outward current ramp (300 pA, 100 ms) into the post-junctional neuron to establish baseline action potential (AP) firing properties. The current ramp injection was repeated during stimulation of the pre-junctional neuron with train of AP inducing current steps (200 Hz, 2 nA, 0.5 ms). This protocol was repeated 100 times. For the second protocol, a 20 ms current step near rheobase (to approximate an average AP firing probability of 0.5) was injected into the post-junctional neuron and repeated 100 times to establish baseline AP firing properties. To examine the impact of pre-junctional activity, the same protocol was performed with simultaneous current steps (200 Hz, 2 nA, 0.3 ms) in pre-junctional neurons.

### Data Analysis

Data analysis was performed using Axograph X software (Berkeley, CA, USA).

Integrated AP firing, calculated from the area under individual input-output (*i-o*) curves was used to compare the firing properties of neurons. AP amplitude, after-hyperpolarization potential (AHP) amplitude and time to AHP peak were all calculated relative to threshold (50 V/s). AP rise-time was calculated as the period between 10% and 90% of maximal AP amplitude. AP half-width was measured at 50% of maximal AP amplitude. The rheobase current was determined as the first current step from which an AP was generated. The input resistance and time constant were calculated in current clamp mode with a current injected to hold the cells at −70 mV. Input resistance was calculated from the voltage deflection relative to baseline that occurred from injection of a −60 pA, 400 ms current step. Time constant was calculated from the voltage decay (1–1/e) that occurred from a −60 pA, 400 ms current injection. Coupling coefficient was calculated as the ratio of the amplitude of the voltage deflection in the post-junctional cell to that in the pre-junctional cell induced by a −60 pA, 100 ms current step. The bidirectional coupling coefficient symmetry was determined by calculating the difference between the coupling coefficients for each of the connected cells.

Membrane potential was determined by calculating the predominate voltage potentials for a period of 500 ms prior to current injection using the histogram function within Axograph X software with a bin width of 0.01 mV. Mean latency to AP firing was calculated from the onset of the current step to the AP threshold for each of the 100 sweeps. Linear fits were made using Prism (GraphPad Software Inc., San Diego, CA, USA).

The distance between electrode tips was determined in a single optical plane using ImageJ software^1^.

### Unsupervised Cluster Analysis

For each IN, the measured electrophysiological features were concatenated into a single vector. These vectors were then used to create a matrix where the columns represented features while rows represented neurons. The values for each feature were normalized by converting into *z*-scores. In order to reduce correlations between features, the principal components of this feature matrix were obtained (Jolliffe, 2002).

Using all the principal component scores unsupervised clustering was performed with Gaussian Mixture Models (GMM; McLachlan and Peel, 2000). The clustering algorithm identified two distinct IN groups. GMM clustering was carried out using the “fitgmdist” function in Matlab 2016 (The MathWorks, Inc., Natick, MA, USA). GMM clustering uses Expectation Maximization (EM) algorithm to find the optimum fit of distributions. Since this is an iterative algorithm, the maximum iteration was set as 1000. GMM uses covariance matrices to describe gaussian distributions. Since PCA scores within an IN group can still be correlated, full covariance matrices were used, which includes correlations between features. The parameter “SharedCovariance” was set to “true” to avoid ill-conditioned covariance matrices. The EM algorithm was run 100 times and the best result (in terms how well the distributions are fitted to the data) was selected. All other parameters in the GMM algorithm were left at their default values.

### Statistical Analysis

GraphPad Prism software (v6; GraphPad Software Inc.) was used for all statistical analysis. Unpaired two-tail Student’s *t*-tests were used to make comparisons between the two INs populations. Paired two-tail Student’s *t*-tests were used to test the effect of pre-junctional activity on the post-junctional cell; including membrane potential, AP count, latency and standard deviation as well as the probability of AP firing. A linear regression analysis was performed to determine the effect of multiple pre-junctional neurons on post-junctional activity with *r*^2^ values reported. In all cases the significance for analysis was set as an alpha value of 0.05. Data are presented as mean ± standard error of the mean (SEM).

## Results

### Electrical Synapses Occur within and between Fast-Spiking and Regular-Spiking INs

**Figure 1:**
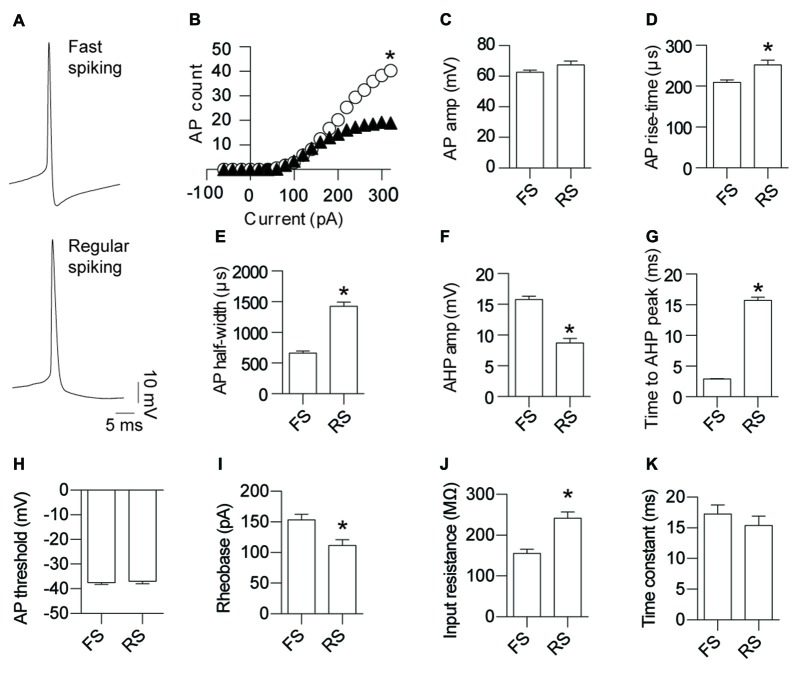
Fast-spiking (FS) and regular-spiking (RS) interneurons (INs) are electrically connected and can be separated into their respective types based on the characteristics of their activity. **(A)** Representative action potential (AP) traces of gap junction coupled layer 5 (L5) cortical GAD67 positive INs: FS and RS. **(B)** Input-output relationship for FS (white circles) and RS (black triangles) neurons (*p* < 0.0012, FS 243.8 ± 21.5, RS 121.5 ± 36.0). Quantification of AP; **(C)** amplitude (*p* = 0.055, FS 62.8 ± 1.19 mV, RS 67.5 ± 2.33 mV); **(D)** rise-time (*p* < 0.0014, FS 209.4 ± 6.46 μs, RS 252.1 ± 11.6 μs); **(E)** half-width (*p* < 0.0001, FS 664.5 ± 31.96 μs, RS 1426 ± 67.68 μs); **(F)** After-hyperpolarization potential (AHP) amplitude (*p* < 0.0001, FS 15.8 ± 0.56 mV, RS 8.7 ± 0.74 mV); **(G)** time to AHP peak (*p* < 0.0001, FS 2.9 ± 0.10 ms, RS 15.7 ± 0.55 ms); **(H)** AP threshold (*p* = 0.73, FS −37.4 ± 0.72 mV, RS −37.0 ± 0.97 mV); **(I)** rheobase (*p* < 0.05, FS 153.1 ± 9.50 pA, RS 111.8 ± 9.40 pA); **(J)** input resistance (*p* < 0.0001, FS 155.1 ± 10.5 MΩ, RS 242.0 ± 15.4 MΩ); and **(K)** time constant (*p* = 0.47, FS 17.3 ± 1.46 ms, RS 15.4 ± 1.54 ms). Data are presented as mean ± SEM. *p < 0.05.

Somatic whole-cell patch-clamp recordings were made from GAD67+ neurons in L5 of the mouse cortex (Figure 1). Using previously reported active and passive neuronal properties to enable manual assignment of cell identity, the recorded cells fell clearly into two classes of INs: FS (79%; Kawaguchi and Kubota, 1993; Galarreta and Hestrin, 1999; Tamás et al., 2004) and RS (21%) IN types (Kawaguchi and Kubota, 1997; Szabadics et al., 2001; Tamás et al., 2004). To objectively determine if our manual classification was robust, we completed an unsupervised cluster analysis of the electrophysiological data. Principal components of the data were determined and subsequent clustering analysis revealed that the recorded neurons fell into two clear populations corresponding with our manual classification (Supplementary Figure S1). In comparison to RS neurons, FS neurons fired significantly more APs (Figure 1B), had similar AP amplitude (Figure 1C), displayed a faster AP rise-time (Figure 1D), narrower AP half-width (Figure 1E), an enhanced AHP amplitude (Figure 1F), a quicker time to peak of AHP (Figure 1G), a larger rheobase (Figure 1I) and a lower input resistance (Figure 1J). No difference was observed for AP threshold (Figure 1H) and time constant (Figure 1K) between FS and RS INs. Importantly, the values we report for these two INs classes are distinct to those reported for neurogliaform cells (Hestrin and Armstrong, 1996; Price et al., 2005; Oláh et al., 2007; Tricoire et al., 2010), strongly suggesting that the INs recorded by us do not belong to this class. The paucity of NGFCs in L5 of the cortex has been reported elsewhere (Oláh et al., 2009; Jiang et al., 2015). Furthermore, the RS INs we describe are also functionally distinct from LTS cells that have previously been reported to form electrical synapses; most notably the cells we define as RS do not fire at the at low stimulation frequencies that define LTS cells (Galarreta and Hestrin, 1999; Gibson et al., 1999; Beierlein et al., 2000; Mancilla et al., 2007).

**Figure 2:**
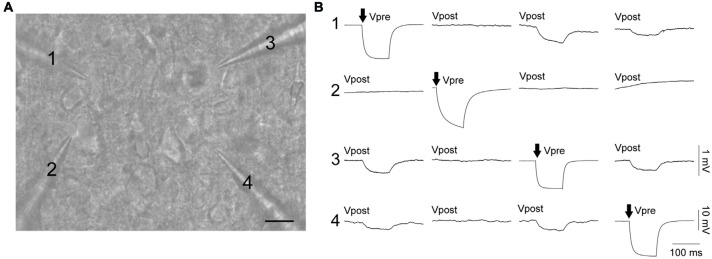
FS and RS INs are connected by gap junctions and form electrical syncytia. **(A)** Oblique infrared image of four INs being patched simultaneously. Scale bar 15 μm. **(B)** Example of a successful quad recording with neurons 1, 3 and 4 reciprocally connected by gap junctions. Both **(A,B)** are from the same data. Arrows represent the cell receiving current injection (−60 pA, 100 ms) in each of the four sets of traces. Vpre indicates the pre-junctional neuron and Vpost the post-junctional neurons. Scale bars are 10 mV for Vpre traces and 1 mV for Vpost traces.

**Figure 3:**
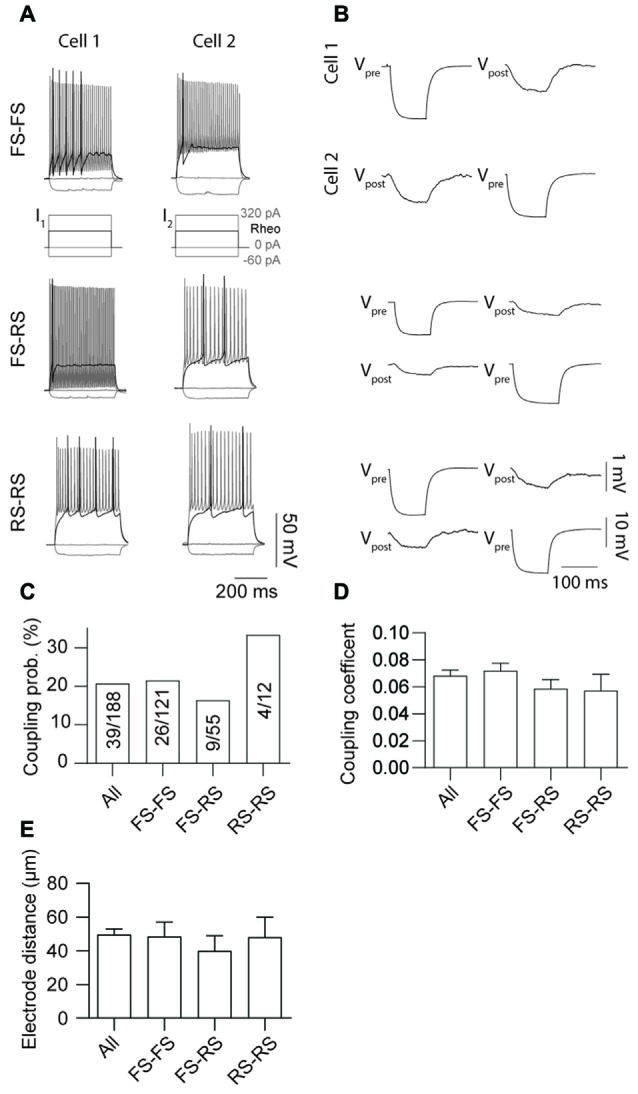
Probability of gap junction coupling is similar within and between different IN classes. **(A,B)** Comparison of AP firing patterns and gap junction connectivity of paired FS to FS (top row), FS to RS (middle row) and RS to RS (bottom row) neurons. In each case Vpre denotes cell that received current injection (−60 pA, 100 ms) and Vpost the post-junctional cells. **(C)** Comparison of occurrence, **(D)** coupling coefficient of gap junction coupling (*p* = 0.52, All 0.068 ± 0.004, FS-FS 0.072 ± 0.006, FS-RS 0.064 ± 0.007, RS-RS 0.041 ± 0.006) and **(E)** distance between electrode tips (*p* = 0.91, All 50.7 ± 3.44 μm, FS-FS 48.45 ± 8.65 μm, FS-RS 39.97 ± 8.97 μm, RS-RS 48.05 ± 11.94 μm) within and between FS and RS neurons. Data are presented as mean ± SEM.

**Figure 4:**
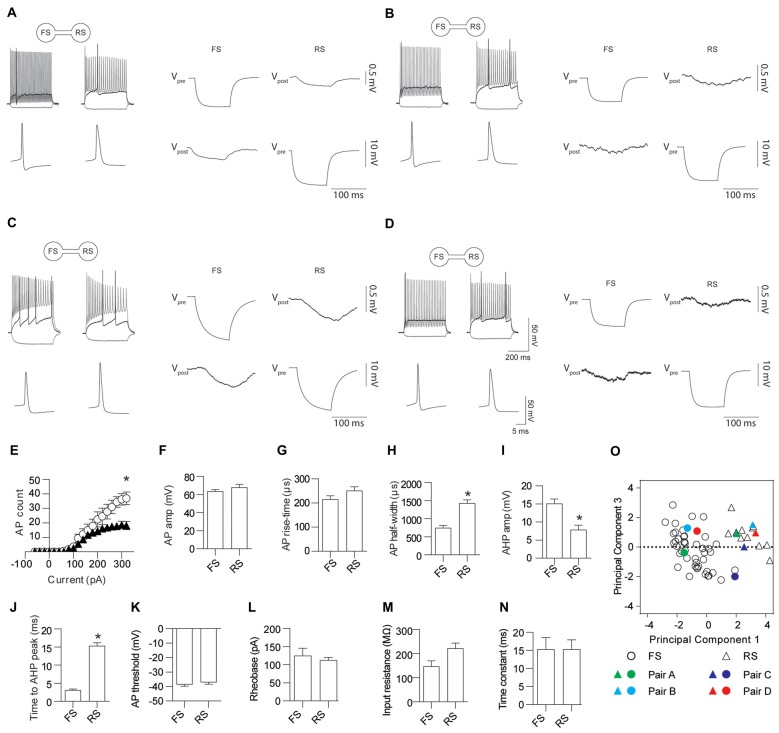
FS and RS INs are gap junction coupled. **(A–D)** Four representative examples of AP firing patterns, the first AP fired and gap junction coupling between FS and RS INs. Quantification of AP firing and kinetics for FS and RS gap junction coupled INs; **(E)** input-output relationship (*p* = 0.023, FS black triangles 226.2 ± 42.4, RS white circles 110.7 ± 17.3), **(F)** amplitude (*p* = 0.30, FS 63.6 ± 1.81 mV, RS 67.8 ± 3.46 mV); **(G)** rise-time (*p* = 0.12, FS 215.5 ± 14.54 μs, RS 251.3 ± 15.72 μs); **(H)** half-width (*p* < 0.0001, FS 750.8 ± 64 μs, RS 1432 ± 91 μs); **(I)** AHP amplitude (*p* = 0.001, FS 15.1 ± 1.24 mV, RS 7.9 ± 1.23 mV); **(J)** time to AHP peak (*p* < 0.0001, FS 3.11 ± 0.32 ms, RS 15.3 ± 0.86 ms); **(K)** AP threshold (*p* = 0.37, FS −38.7 ± 1.07 mV, RS −37.1 ± 1.33 mV); **(L)** rheobase (*p* = 0.57, FS 125.0 ± 20.27 pA, RS 112.5 ± 7.50 pA); **(M)** input resistance (*p* = 0.034, FS 146.8 ± 22.91 MΩ, RS 221.5 ± 22.01 MΩ); and **(N)** time constant (*p* = 0.99, FS 15.3 ± 3.22 ms, RS 15.4 ± 2.57 ms). **(O)** Principle component analysis and unsupervised clustering of recorded neurons demonstrating where the FS (circles) and RS (triangles) INs shown in **(A–D)** lie within the entire dataset. Vpre indicates the pre-junctional neuron and Vpost the post-junctional neurons. Data are presented as mean ± SEM. *p < 0.05.

Electrical coupling within and between FS and RS neurons was investigated using quad patch-clamp recordings. The average distance between the electrode tips during recordings was 50.8 ± 3.32 μm (Figure 2A), similar to previous reports (Galarreta and Hestrin, 1999, 2002; Gibson et al., 2005). Figure 2B illustrates a successful quad recording showing electrical coupling between three neurons. A total of 72 quad recording attempts were made with a success rate of 14 quads, 23 trios and 35 duos. From these 188 opportunities for observing electrically coupled neurons, 39 pairs were found (Figure 3). Therefore, the probability of electrical coupling of GAD67+ neurons within a 100 μm region in a slice with a thickness of 300 μm of cortical L5 was 21%. Electrical coupling was detected between FS to FS, RS to RS, and FS to RS neurons (Figures 3A,B). All traces of coupled FS-RS neurons can be seen in Figures 4A–D and Supplementary Figure S2 and the quantification of these INs in Figures 4E–N. No difference between the probability of coupling and the coupling coefficient within or between the FS and RS classes or the distance between any of the coupling types was observed (Figures 3C–E). There was also no difference between the bidirectional coupling coefficient symmetry between the three coupling types (*p* = 0.75, FS-FS 0.022 ± 0.0045, FS-RS 0.026 ± 0.0070, RS-RS 0.016 ± 0.0075) or the ratio of the coupling coefficients (*p* = 0.58, FS-FS 1.2 ± 0.25, FS-RS 0.7 ± 0.15, RS-RS 1.3 ± 0.35). These data suggest that the efficacy of electrical coupling is also similar within and between these two functional IN subclasses.

### Pre-Junctional Current from a Single IN Enhances Post-Junctional Excitability

**Figure 5:**
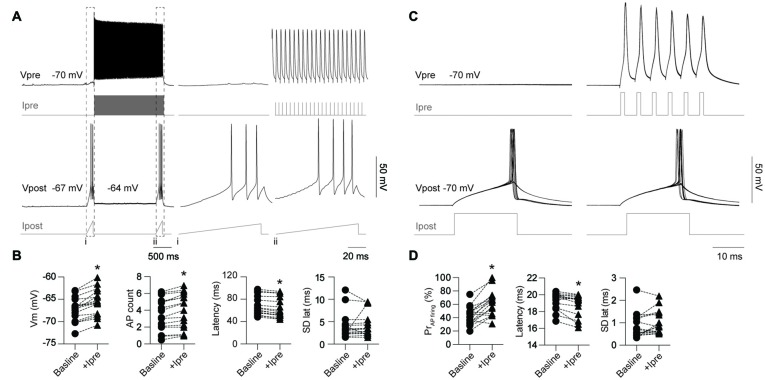
Activity in a single pre-junctional neuron increases the excitability of a post-junctional IN. **(A)** Examples of APs in the pre-junctional neuron (Vpre) increasing the excitability of the post-junctional neuron (Vpost) as revealed by injection of outward currents steps in the pre-junctional (Ipre) and post-junctional neuron (Ipost). Note: (i) absence and (ii), presence of pre-junctional AP firing. Dashed box indicates of the location of the inset from the full trace. Right panels are expanded views of traces on the left, note different scale bar. **(B)** Pooled data showing significant effect of pre-junctional activity on post-junctional voltage (Vm; *p* < 0.0001, baseline −67.2 ± 0.63 mV, +Ipre −65.6 ± 0.69 mV), AP count (*p* < 0.0001, baseline 3.5 ± 0.48, +Ipre 3.98 ± 0.52), latency (*p* < 0.0001, baseline 67.9 ± 4.17 ms, +Ipre 63.8 ± 4.42 ms) and standard deviation of AP latency (*p* = 0.70, baseline 3.8 ± 0.31 ms, +Ipre 3.15 ± 0.33 ms). **(C)** Comparison of post-junctional AP firing near rheobase in presence and absence of pre-junctional activity (10 sweeps shown in each case). **(D)** Pooled data showing significant effect of pre-junctional activity on post-junctional AP firing including AP firing probability (*p* < 0.0001, baseline 42.8 ± 3.42%, +Ipre 62.1 ± 4.75%), AP firing latency (*p* < 0.01, baseline 19.3 ± 0.25 ms, +Ipre 18.9 ± 0.33 ms) and standard deviation of AP latency (*p* = 0.07, baseline 0.86 ± 0.14 ms, +Ipre 0.99 ± 0.13 ms). *n* = 16 for all paired comparisons. Data are presented as paired individual points. *p < 0.05.

Previous reports have demonstrated that electrical coupling enhances neuronal excitability by providing a source of inward current in the post-junctional cell during pre-junctional cell firing leading to synchronize spontaneous AP firing between the connected cells (Galarreta and Hestrin, 1999; Gibson et al., 1999; Mann-Metzer and Yarom, 1999; Tamás et al., 2000; Hu and Agmon, 2015). Here we further investigate the impact of gap junctions on excitability by testing the impact of firing in a single pre-junctional cell on the probability of AP generation in the post-junction cell using two different protocols. In order to investigate the maximum impact a single pre-junctional neuron can have on its partner, outward current test ramps were injected in the post-junctional cell while simultaneously determining baseline AP latency and count (Figures 5A,B). Following this ramp a second test ramp was delivered but on this occasion the pre-junctional cell was stimulated with a train of brief depolarizing current steps to reliably trigger APs (Figure 5A), and this protocol was repeated 100 times. All the following experiments were conducted in the presence of the AMPA receptor antagonist CNQX (20 μM) to isolate the post-junctional response resulting from current passing through the gap junctions. This protocol was performed on nine FS-FS pairs, two FS-RS pairs and three RS-RS pairs, which were grouped for later analysis. Pre-junctional activity resulted in a highly reproducible depolarization in the post-junctional neuron, an increase in the total number of APs during the test ramp and a reduction in the latency to the first AP without altering the precision of firing (Figure 5B).

A second protocol that examines modulation of excitability near rheobase was also used to investigate the impact of pre-junctional activity. The stimulating current in the post-junctional neuron was set near to rheobase such that APs fired around 50% of the time to allow for positive or negative modulation by pre-junctional activity. One-hundred sweeps were also used for this protocol to determine firing probability (Figure 5C) in the presence of CNQX. This was performed on five FS-FS and three FS-RS pairings. Burst firing in the pre-junctional neuron significantly increased firing probability and reduced latency to first AP with no impact on the precision of firing (Figure 5D). These results show that pre-junctional current from a single neuron can readily modulate the excitability of its partner and that this occurs irrespective of the functional subclass of the IN.

### Simultaneous Activation of Two Pre-Junctional Neurons has an Additive Effect on Post-Junctional Excitability

**Figure 6:**
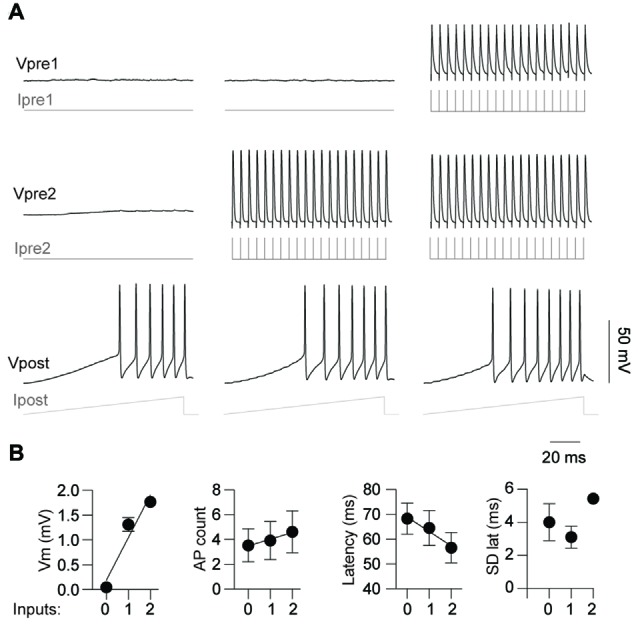
Simultaneous activation of two pre-junctional neurons has an additive effect on post-junctional excitability.** (A)** Representative traces from a recording of three-gap junction coupled neurons demonstrating increasing excitability with simultaneous pre-junctional activity. **(B)** Pooled data showing additive effect of pre-junctional activity on Vm (*r*^2^ = 0.93), AP count (*r*^2^ = 0.97), AP latency (*r*^2^ = 0.96) and standard deviation of AP latency (*r*^2^ = 0.16); 0, 1 and 2 denote the amount of simultaneous pre-junctionally active neurons. *n* = 3 for regression analyses. Data are presented as mean ± SEM.

Having demonstrated that activity from a single pre-junctional cell is sufficient to increase AP firing in a post-junctional cell, we next investigated how activity in multiple pre-junctional cells alters the post-junctional excitability. Previous reports show that a single IN couples to up to nine other INs by gap junctions (Peinado et al., 1993; Mann-Metzer and Yarom, 1999; Fukuda, 2017), indicating that each coupled cell has many pre-junctional partners. However, it is currently unknown how the collective activity of multiple neurons affects their post-junctional counterpart. On three occasions, three mutually coupled neurons were recorded thus permitting the analysis of the impact of dual simultaneous inputs on post-junctional neuron excitability (Figure 6). These three cases of coupling were composed of a triplet of FS INs, a triplet of RS INs as well as two RS and a single FS IN. Injection of a current ramp in the absence of pre-junctional inputs elicited a reproducible train of APs (Figure 6A, left). Consistent with the data above, activation of a single pre-junctional neuron produced a depolarizing shift, increased AP firing and decreased latency to first AP in the post-junctional neuron without affecting the precision of firing (Figures 6A,B, middle). Interestingly, the simultaneous activation of a second pre-junctional neuron produced: (i) a larger depolarizing shift in the post-junctional membrane potential; (ii) more APs, as well as; and (iii) a further reduction in the latency of AP firing without altering firing precision (Figures 6A,B, right). Furthermore, a strong linear relationship between post-junctional membrane potential, AP count and latency and the number of pre-junctional neurons was observed. These data demonstrate that there is an additive effect of activity in pre-junctional neurons that occurs regardless of IN subclass.

## Discussion

A key finding of this study is that gap junctions couple L5 INs of different functional classes with the same probability as those of the same class. This observation has significant implications on the role of gap junctions in controlling cortical network activity. Here we categorize INs into either FS or RS based on their firing properties (Kawaguchi and Kubota, 1993; Tamás et al., 2004; Simon et al., 2005; Oláh et al., 2007). Although there is the potential to further sub-classify using features such as morphology and protein expression, it is generally recognized that these two broad classes underlie very distinct functions within cortical networks. FS neurons for example drive gamma oscillations which are critical for several physiological processes including attention, perception and working memory (Singer and Gray, 1995; Jefferys et al., 1996; Siegel et al., 2009; Sohal et al., 2009). Conversely, RS neurons are involved in the generation of beta (12–30 Hz) frequency activity that is related to voluntary controlled sensorimotor actions (Salmelin et al., 1995; Szabadics et al., 2001; Tamás et al., 2004; Roopun et al., 2006).

With the exception of neurogliaform cells, INs from the same class have previously been reported to predominantly form gap junction connected networks composed of cells from the same functional subclass (Galarreta and Hestrin, 1999; Gibson et al., 1999; Tamás et al., 2000; Venance et al., 2000). For example, Galarreta and Hestrin (1999) described gap junction coupling exclusively between FS neurons in L5 of the somatosensory and visual cortices. However, while there have been reports of different INs coupling in small numbers (Gibson et al., 1999; Caputi et al., 2009), our results challenge the predominant view, and we report that probabilities of gap junction connectivity within and between functionally distinct IN subclasses are similar within L5 cortical somatosensory networks.

The lack of previous studies demonstrating abundant coupling between different functional IN subclasses may be explained by sampling bias. Previous reports have typically based coupling frequency estimates on sample sizes that are relatively small, with an average of 40 possible pairings reported (Galarreta and Hestrin, 1999, 2002; Gibson et al., 1999; Venance et al., 2000). This is contrasted by the present study where 188 possible pairings were recorded. The previous reports are therefore more likely to display a sampling bias towards INs that are commonly found within a particular brain area. In particular, FS PV^+^ basket cells are common in L5 of the cortex and have a readily identifiable morphology in electrophysiological experiments, which may contribute to why they comprise the main IN subclass that has been described to be connected by gap junctions (Galarreta and Hestrin, 1999, 2002; Gibson et al., 1999; Venance et al., 2000; Oláh et al., 2007; Xu et al., 2010; Hu and Agmon, 2015).

While current estimates of the number of neurons coupled via gap junctions vary and are developmentally regulated, dye coupling experiments have reported that a single IN couples to approximately eight others (Peinado et al., 1993; Mann-Metzer and Yarom, 1999). A striking observation in the present study is that, despite the small effect of a single gap junction on post-junctional resting membrane potential, there were clear effects on the firing probability of the post-junctional neuron as well as the previously reported ability of gap junction mediated activity to synchronize firing (Galarreta and Hestrin, 1999, 2002; Gibson et al., 1999; Hu and Agmon, 2015). In view of the incremental effects of dual and triple connections seen in parallel recordings of multiple INs, it is obvious that during network events *in vivo*, the effects of a large number of simultaneously active gap junctions will have a profound impact on a post-junctional neuron. While not observed in the current study due to pharmacological antagonism, previous reports have demonstrated that INs can be coupled by both electrical and chemical synapses to create complicated voltage responses in the post-synaptic cell even when the two neurons are from the same functional subclass (Galarreta and Hestrin, 1999, 2002; Tamás et al., 2000; Hu and Agmon, 2015).

Gap junction connectivity is developmentally regulated with the number of coupled cells decreasing with age (Peinado et al., 1993; Rörig et al., 1995). Interestingly, the age range where large numbers of neurons are connected by gap junctions in rodents is early in postnatal development, a time period where synchronous neuronal activity is important for the maturation of neuronal circuits (Zhang and Poo, 2001). Gap junctions are therefore well positioned to influence neuronal excitability during brain development that could have marked long term effects on neurological disorders such as epilepsy, autism and schizophrenia (Lewis et al., 2005; Welsh et al., 2005; Volman et al., 2011). In support of these ideas, modulation of gap junctions through blockade or genetic manipulation alter network excitability and seizure susceptibility (Nassiri-Asl et al., 2009; Voss et al., 2009; Jacobson et al., 2010; Medina-Ceja and Ventura-Mejía, 2010). However, due to the developmental regulation of the number of cells coupled by gap junctions, an interesting question for future work is whether the coupling probabilities we describe here are also developmentally regulated.

In conclusion, our results demonstrate that gap junction coupling is common across neocortical INs of different firing subclasses. This observation will expand current concepts on the functional repertoire of neuronal networks to include synchronous firing of functionally distinct INs.

## Author Contributions

RJH, KK, CAR and SP designed and conceptualized the study. RJH performed all experiments and data analysis. GDCM conducted principal component analysis. RJH, GDCM, KK, CAR and SP wrote the manuscript. All authors have approved the final version of this manuscript, agree to be accountable for all aspects of the work and qualify for authorship.

## Conflict of Interest Statement

The authors declare that the research was conducted in the absence of any commercial or financial relationships that could be construed as a potential conflict of interest.

## Footnotes

^1^https://imagej.nih.gov/ij/

## Supplementary Material

FIGURE S1 Principle component analysis and unsupervised clustering of recorded interneurons (INs). Graphs depict the first four principles components and the unsupervised clustering groupings from each comparison for cells classified as fast-spiking (FS; open circles) or regular-spiking (RS; closed triangles).

FIGURE S2 Action potential (AP) firing patterns of FS-RS pairings. **(A–C)** Depicts the AP firing patterns, the first AP fired and gap junction coupling between the remaining FS and RS INs pairings not shown in Figure 4. Vpre indicates the pre-junctional neuron and Vpost the post-junctional neurons. **(D)** Principle component analysis and unsupervised clustering of recorded neurons demonstrating where the FS (circles) and RS (triangles) INs shown in **(A–C)** lie within the entire dataset.
