# Supplementary figures and images for: Gap Junctions Link Regular-Spiking and Fast-Spiking Interneurons in Layer 5 Somatosensory Cortex
Source: Front Cell Neurosci. 2017 Jul 17;11:204. doi: 10.3389/fncel.2017.00204 (PMC5511827; doi:10.3389/fncel.2017.00204)

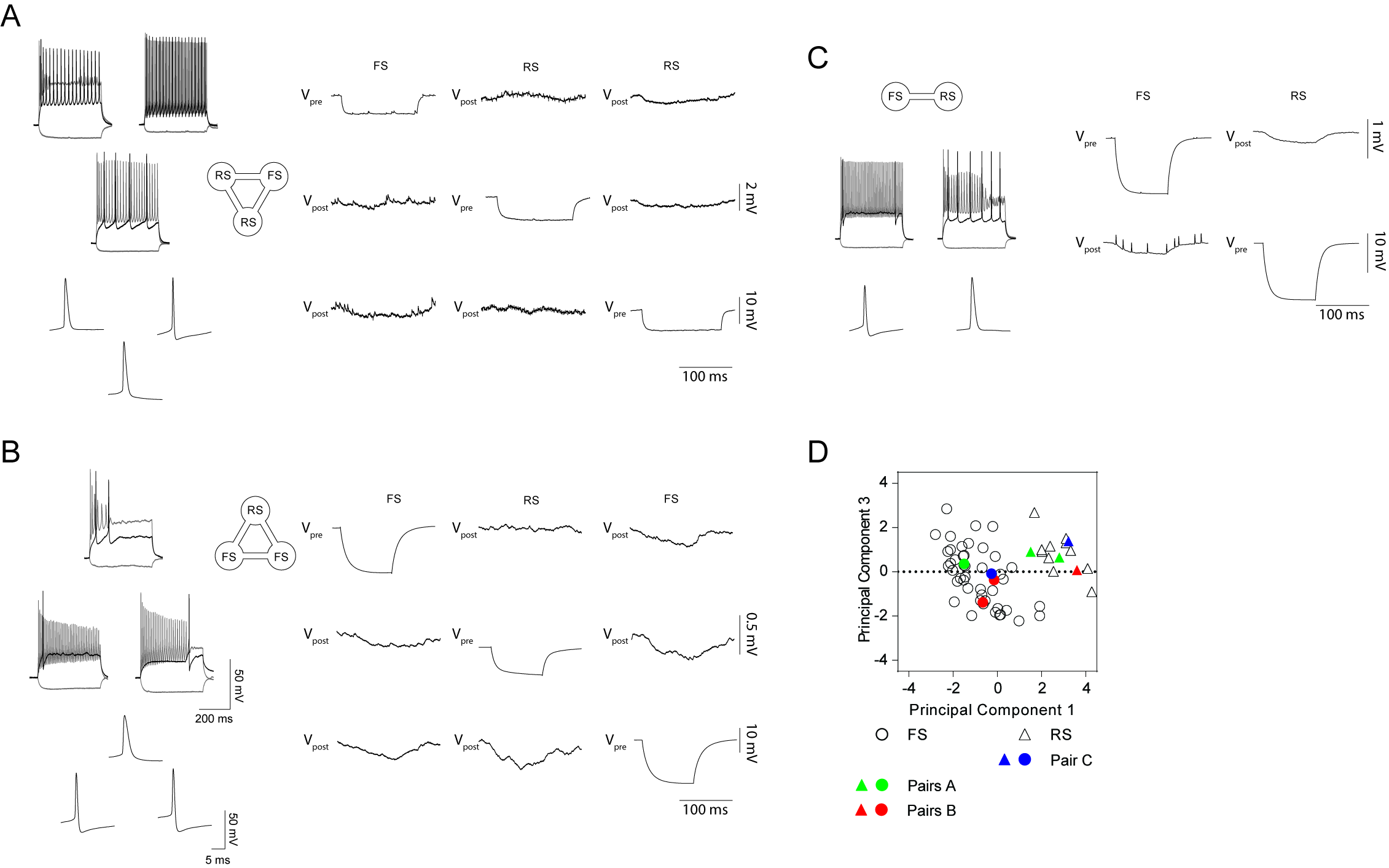

Supplement: FIGURE S2 — Action potential (AP) firing patterns of FS-RS pairings. (A–C) Depicts the AP firing patterns, the first AP fired and gap junction coupling between the remaining FS and RS INs pairings not shown in Figure 4. Vpre indicates the pre-junctional neuron and Vpost the post-junctional neurons. (D) Principle component analysis and unsupervised clustering of recorded neurons demonstrating where the FS (circles) and RS (triangles) INs shown in (A–C) lie within the entire dataset. [file Image_2.tif]
